# Supplementary material for: Lagged effect of temperature and rainfall on malaria incidence in Colombia (2013–2023): An approach with Bayesian spatiotemporal adjustment
Source: PLOS Glob Public Health. 2026 Mar 9;6(3):e0006104. doi: 10.1371/journal.pgph.0006104 (PMC12970859; doi:10.1371/journal.pgph.0006104)

**S1 Fig**

Histogram of the frequency of weekly temperature and rainfall in Colombian municipalities with altitudinal suitability for malaria transmission.


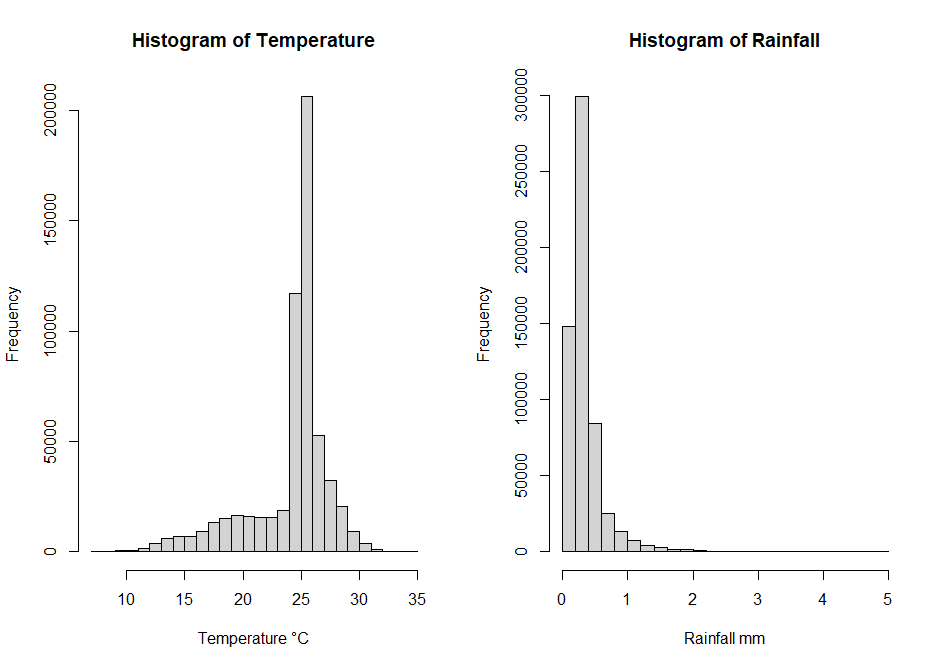

Supplement: S1 Fig — (DOCX) [file pgph.0006104.s001.docx]
