# Supplementary material for: Lagged effect of temperature and rainfall on malaria incidence in Colombia (2013–2023): An approach with Bayesian spatiotemporal adjustment
Source: PLOS Glob Public Health. 2026 Mar 9;6(3):e0006104. doi: 10.1371/journal.pgph.0006104 (PMC12970859; doi:10.1371/journal.pgph.0006104)
Supplement: Attachment — Submitted filename: Response to Reviewers.docx [file pgph.0006104.s003.docx]

Journal Requirements:

1. We note that you have included your Figures within the body of your manuscript. Please remove the Figures from the body of your manuscript and upload them as separate Figure files.

R: We accept the comment and we removed the Figures from the body of the manuscript.

2. We have noticed that you have uploaded Supporting Information files, but you have not included a list of legends. Please add a full list of legends for your Supporting Information files after the references list.

R: We accept the comment and we modified the Supplements legend section (pg. 34).

If the reviewer comments include a recommendation to cite specific previously published works, please review and evaluate these publications to determine whether they are relevant and should be cited. There is no requirement to cite these works unless the editor has indicated otherwise.

**Comments to the Author**

1. Does this manuscript meet PLOS Global Public Health’s [publication criteria](https://track.editorialmanager.com/CL0/https:%2F%2Fjournals.plos.org%2Fglobalpublichealth%2Fs%2Fcriteria-for-publication/1/010f019c02149c30-34ae9f8b-1dfa-4a53-b844-8e3686e02d17-000000/yFtE7nsNHF8q0F7hb3ME40X6zuKKkObdY8c5pJBhDKQ=247)? Is the manuscript technically sound, and do the data support the conclusions? The manuscript must describe methodologically and ethically rigorous research with conclusions that are appropriately drawn based on the data presented.

Reviewer #1: Partly

2. Has the statistical analysis been performed appropriately and rigorously?

Reviewer #1: I don't know

3. Have the authors made all data underlying the findings in their manuscript fully available (please refer to the Data Availability Statement at the start of the manuscript PDF file)?

The [PLOS Data policy](https://track.editorialmanager.com/CL0/https:%2F%2Fjournals.plos.org%2Fglobalpublichealth%2Fs%2Fdata-availability/1/010f019c02149c30-34ae9f8b-1dfa-4a53-b844-8e3686e02d17-000000/_1cl3nZwwp6CfvyWSVop0EwQRLzgk0MC-ue8MP_gl18=247) requires authors to make all data underlying the findings described in their manuscript fully available without restriction, with rare exception. The data should be provided as part of the manuscript or its supporting information, or deposited to a public repository. For example, in addition to summary statistics, the data points behind means, medians and variance measures should be available. If there are restrictions on publicly sharing data—e.g. participant privacy or use of data from a third party—those must be specified.

Reviewer #1: No

4. Is the manuscript presented in an intelligible fashion and written in standard English?

PLOS Global Public Health does not copyedit accepted manuscripts, so the language in submitted articles must be clear, correct, and unambiguous. Any typographical or grammatical errors should be corrected at revision, so please note any specific errors here.

Reviewer #1: Yes

5. Review Comments to the Author

Please use the space provided to explain your answers to the questions above. You may also include additional comments for the author, including concerns about dual publication, research ethics, or publication ethics. (Please upload your review as an attachment if it exceeds 20,000 characters)

Reviewer #1:

This is a well written article on an important topic given the potential for the rise in Malaria cases due to changes in the climate. However, I have several suggestions and questions that I hope can make the article stronger:

Line 150: “We grouped the daily data by week…” Authors state that the daily temperatures data were grouped. Authors should clarify if valued were aggregated, averaged, etc.

R: We appreciate the comment, and we estimated the weekly averages of temperature and rainfall for each municipality, as mentioned in lines 151-153.

Lines 181-184: Authors should add a note regarding the justification of their choices for the knots for temperature and rainfall (especially since the percentiles are different). Also, there should a be a note regarding the number of knots.

R: We accept the comment, and we have included a couple of paragraphs regarding the justification of the choices for the knots (lines 188-204).

Lines 195-196: Authors should clarify the percentage of zeros in the data and consequently, justify the need for a zero-inflated model. At a minimum authors should report model comparison results between their model choice and a regular negative binomial model based on measures like DIC and WAIC.

R: We accept the comment, and we have modified the methods to compare two models a zero-inflated negative binomial model vs a regular negative binomial model. We selected the best of the two models based on the Logarithmic Conditional Predictive Ordinate (LCPO), Watanabe–Akaike Information Criterion (WAIC), and Deviance Information Criterion (DIC) (lines 212-218). We observed that the negative binomial model showed better performance and used this model for the estimation (lines 269-278).

Authors do not provide any measures of model fit or residual analysis to confirm the model they fitted to the data is an adequate model.

R: We accept the comment, and we have included the diagnostic plots for the temperature and rainfall negative binomial models (lines 279-299).

Minor comments:

Line 138: “on February 21, 2025” not necessary

R: We appreciate the comment; however, for retrospective studies, the journal requires authors to indicate the day, month, and year when the data were accessed for research purposes.
